# The Anti-Oxidant Curcumin Solubilized as Oil-in-Water Nanoemulsions or Chitosan Nanocapsules Effectively Reduces *Helicobacter pylori* Growth, Bacterial Biofilm Formation, Gastric Cell Adhesion and Internalization

**DOI:** 10.3390/antiox12101866

**Published:** 2023-10-16

**Authors:** Antonio Hidalgo, Denisse Bravo, Cristopher Soto, Gabriela Maturana, Jimena Cordero-Machuca, María Carolina Zúñiga-López, Felipe Oyarzun-Ampuero, Andrew F. G. Quest

**Affiliations:** 1 Cellular Communication Laboratory, Center for Studies on Exercise, Metabolism and Cancer (CEMC), Faculty of Medicine, Universidad de Chile, Santiago 8380453, Chile; ntn.hidalgo@gmail.com (A.H.); chrissoto@ug.uchile.cl (C.S.); 2 Advanced Center for Chronic Diseases (ACCDiS), Universidad de Chile, Santos Dumont 964, Independencia, Santiago 8380494, Chile; denisse.bravo@unab.cl (D.B.); jimena.cordero@ug.uchile.cl (J.C.-M.); 3 Cellular Interactions Laboratory, Faculty of Dentistry, Universidad Andrés Bello, Santiago 8370133, Chile; 4 Department of Inorganic and Analytical Chemistry, Faculty of Chemical and Pharmaceutical Sciences, Universidad de Chile, Santiago 8380494, Chile; gamaturana@uchile.cl (G.M.); mczuniga@ciq.uchile.cl (M.C.Z.-L.); 5 Departament of Sciences and Pharmaceutical Technology, Faculty of Chemical and Pharmaceutical Sciences, Universidad de Chile, Santiago 8380494, Chile

**Keywords:** curcumin, nanoemulsions, nanocapsules, *Helicobacter pylori*, toxicity, gastric cancer

## Abstract

The bacterium *Helicobacter pylori* (*H. pylori*) represents a major risk factor associated with the development of gastric cancer. The anti-oxidant curcumin has been ascribed many benefits to human health, including bactericidal effects. However, these effects are poorly reproducible because the molecule is extremely unstable and water insoluble. Here we solubilized curcumin as either nanoemulsions or chitosan nanocapsules and tested the effects on *H. pylori*. The nanoemulsions were on average 200 nm in diameter with a PdI ≤ 0.16 and a negative zeta potential (−54 mV), while the nanocapsules were 305 nm in diameter with a PdI ≤ 0.29 and a positive zeta potential (+68 mV). Nanocapsules were safer than nanoemulsions when testing effects on the viability of GES-1 gastric cells. Also, nanocapsules were more efficient than nanoemulsions at inhibiting *H. pylori* growth (minimal inhibitory concentration: 50 and 75 μM, respectively), whereby chitosan contributed to this activity. Importantly, both formulations effectively diminished *H. pylori’s* adherence to and internalization by GES-1 cells, as well as biofilm formation. In summary, the demonstrated activity of the curcumin nanoformulations described here against *H. pylori* posit them as having great potential to treat or complement other therapies currently in use against *H. pylori* infection.

## 1. Introduction

Cancer is the second leading cause of deaths worldwide, and among the different types of cancer, gastric cancer is considered to have one of the highest mortality rates and the worst prognosis after diagnosis [1]. Various environmental and nutritional factors are involved in the development of this disease, including sedentary lifestyle, poor diet, tobacco and/or alcohol consumption, stress, and family history, among others. Moreover, several studies showed that some gastric cancers are not associated with these factors and that infection with pathogens (viruses or bacteria) can favor the development of this disease [2]. Indeed, one of the main risk factors associated with the development of gastric cancer is the presence of *Helicobacter pylori* (*H. pylori*) in the stomach [3]. *H. pylori* is a Gram-negative microaerophilic bacterium with helical-shaped flagella that particularly infects the gastric epithelium. In humans, it can be detected in more than 50% of the world population [4].

*H. pylori* is able to attach to and internalize into gastric epithelial cells. Several virulence factors permit the adherence to the membrane of epithelial cells, such as BabA, SabA, OipA, and HopQ, among others [5]. After attachment, *H. pylori* can translocate proteins from the bacteria into the cytoplasm of infected cells, through a mechanism involving the multi-component bacterial type IV secretion system (T4SS) and the protein CagA [6]. These components are encoded in the Cag-pathogenicity island (*cagPAI*), which is essential for T4SS function and CagA traslocation [6]. Once inside the host cells, *H. pylori* virulence factors increase the survival and proliferation rate of infected cells [7] and induce the production of pro-inflammatory cytokines, such as IL-8 [8], which promote cell transformation.

*H. pylori* persists as a chronic infection unless treated, likely due to the presence of survival mechanisms that permit adaption to the acidic stomach environment and the formation of biofilms [9]. The latter ability is important, because it reduces the efficiency of antibiotic therapy and the possibility of clearance by the host immune response [9]. In particular, biofilm-associated *H. pylori* have been shown to be more resistant in vitro to clarithromycin, one of the antibiotics commonly used to treat *H. pylori* infections. The authors noted a four-fold increase in the minimal inhibitory concentration (MIC) to prevent bacterial growth in biofilms, as compared to bacteria growing individually [10]. Additionally, an increased mutation rate was observed for biofilm-associated *H. pylori*, which in turn facilitates the development of clarithromycin-resistant strains [10].

To eradicate this bacterium, various antibiotics are used following standard regimens, referred to as triple and quadruple therapy [11]. Antibiotics employed in this context include amoxicillin, clarithromycin, and metronidazole, which in different combinations result in greater than 70% efficiency of eradication [12]. Subsequently, if this does not suffice to eliminate *H. pylori*, the patient is given quadruple therapy, which includes the use of a proton pump inhibitor (PPI), bismuth, metronidazole, and tetracycline, also for 14 days. Although this treatment currently represents the gold standard, its effectiveness has been seen diminished by factors such as resistance to antibiotics (due to the indiscriminate use of these substances) and because people do not apply the treatment protocol rigorously enough [13]. Indeed, it has been estimated that the recurrence rate worldwide after treatment is around 4.3% [14].

Several options are being considered to improve the efficacy of treatments against *H. pylori* infection and thereby prevent the possible long-term development of cancer. Some of these have been developed using pharmaceutical and nanotechnological approaches, whereby the basic idea is to generate formulations that either preserve antibiotic function over extended periods of time [15] or involve generating formulations including new molecules with antibacterial effects [16].

A potentially attractive candidate, in the latter case, is curcumin, a polyphenolic anti-oxidant molecule (formula: 1,7-bis (4-hydroxy-3-methoxyphenyl)-1,6-heptadiene-3,5-dione), found in plants growing in India and Southeast Asia [17]. For centuries, these plants and their alcoholic extracts have been used for medicinal purposes, because of their antibacterial [18] and healing (favoring the formation of an extracellular matrix) [19] properties. In addition, curcumin has been shown to have anti-cancer and anti-inflammatory properties [20], as well as act as an antioxidant [21], amongst others. However, in clinical trials, it has been reported that at least 12 g/day of curcumin is required to obtain any notable therapeutic effects [22], essentially due to the low water solubility and bioavailability of this active molecule [23]. Therefore, when this molecule is consumed orally, only a small fraction is absorbed by the intestine and much of it is rapidly metabolized (about 75%), mainly via sulfation and glucuronidation, to be subsequently eliminated in the feces and urine [24]. In addition, curcumin is extremely sensitive to light and oxygen [25], and, importantly, exposure to either significantly reduces the concentration of the bioactive compound. Ideally, therefore, a formulation is required that permits simultaneous solubilization, dispersing in aqueous/biological medium and stabilizing the curcumin in order to develop its maximum therapeutic potential [26]. Several examples of nanotechnological formulations containing curcumin are available which permit exploiting its beneficial effects in different settings [27]. Among them, the combination of curcumin and other natural agents, such as garlic extract, have been evaluated. Using this formulation, strong anti-inflammatory effects were observed in vivo in diabetic rats as a model [28]. Alternatively, another study showed that chitosan/curcumin nanoparticles had strong cytotoxic effects in vitro in colorectal cancer cells [29].

The antimicrobial effects of curcumin have been observed for a wide variety of bacterial species. Using curcumin included by poly(lactic-co-glycolic acid (PLGA)-nanocapsules, antimicrobial effects were reported for *Staphylococcus aureus* (Gram-positive), *Bacillius subtilis* (Gram-positive), *Escherichia coli* (Gram-negative), and *Pseudomonas aeruginosa* (Gram-negative) [17]. In addition, in vivo approaches showed that curcumin nanoparticles coated by chitosan-tripolyphosphate displayed high antimicrobial activity (over 75%) against *Staphylococcus aureus* and *Pseudomonas aeruginosa* infections [30]. For *H. pylori*, the antimicrobial effect of curcumin was demonstrated, in vitro and in vivo, using clinical isolates from Indian patients [31]. Furthermore, curcumin was shown to reduce the inflammation induced by *H. pylori* in infected mice, as well as the biofilm formation ability [32]. A similar effect on biofilm formation was observed for curcumin together with a decrease in the adherence to gastric Hep-2 cells [33].

In previous studies, we showed that curcumin could be solubilized and stabilized as a nanoemulsion, with potent cancer cell cytotoxicity in vitro and the ability in vivo, in a mouse melanoma model, to prevent re-incident and metastatic tumor growth [34]. These studies indicated that such nanoformulations, including curcumin, had tremendous potential for biomedical applications. However, whether they would serve to eliminate *H. pylori* was not clear. In our current study, we compared the previously described nanoemulsion of curcumin with a chitosan-based nanocapsule in terms of their ability to solubilize curcumin, dispersing the molecule in aqueous/biological media, and elicit bacteriostatic and bacteriotoxic effects towards *H. pylori*. We observed that the nanoencapsulated presentation of curcumin was more effective in reducing *H. pylori* growth, adhesion to gastric cells, bacterial internalization, and biofilm formation.

## 2. Materials and Methods

### 2.1. Materials

#### 2.1.1. Nanocarriers

Curcumin (from curcuma longa, CAS# 458-37-7, purity 75%, product number C1386, Sigma-Aldrich, Burlington, MA, USA), low-molecular-weight chitosan (CAS# 9012-76-4, 84% deacetylation, 94 cps, product number 448869, Sigma-Aldrich, USA), Miglyol 812 (neutral oil formed by esterification of caprylic fatty acids, CAS# 73398-61-5, 57.2% of C8 fatty acids and 42.4% of C10 fatty acids, Sasol, Houston, TX, USA), Epikuron 145 V (soy lecithin fraction enriched with phosphatidylcholine, CAS 8002-43-5, ≥97% phospholipids, Cargill, Düsseldorf, Germany), acetone, and ethanol were from Merck (Darmstadt, Germany).

#### 2.1.2. Scanning Transmission Electron Microscopy (STEM) Images

Phosphotungstic acid and Copper grid 300Mesh Formvar/Carbon 25/pk were from Sigma-Aldrich (USA).

#### 2.1.3. Cell Studies

Cell medium and antibiotics (RPMI 1640) were from Gibco-BRL (Billings, MT, USA). Fetal bovine serum (FBS) was from Biological Industries (Cromwell, CT, USA). The 3-(4,5-dimethylthiazole-2-yl)-5-(3-carboxymetoxyphenyl)-2-(4-sulfophenyl)-2H internal tetrazolium was from Promega (Madison, WI, USA). The CellTiter 96^®^ AQueous One Solution Cell Proliferation Assay (MTS) was from Promega (USA). Gentamycin was from US biological (Detrick, MD, USA). Trypsin-EDTA was from Hyclone (Logan, UT, USA). Phosphate-buffered saline (PBS) and penicillin-streptomycin were from Gibco BRL (USA). Trypan blue and sodium dodecylsulfate (SDS) were from Winkler ltda. (Santiago, Chile).

### 2.2. H. pylori Culture

The reference strain *H. pylori* 26695 (ATCC 700392) cagA+ vacAs1m1 was used. This bacterium was grown as previously described [35] on blood agar plates supplemented with 5% horse serum from Biological Industries (Cromwell, Connecticut USA) and the antibiotic supplement Dent Oxoid (Hampshire, UK) for 16–20 h at 37 °C in a humidified atmosphere with 5% CO_2_.

### 2.3. Cell Culture

GES-1 gastric cells, an immortalized cell line from the normal gastric epithelium (RRID: CVCL_EQ22, Universidad Católica de Chile), were maintained as monolayer cultures in RPMI1640 medium supplemented with 10% FBS and 1% antibiotics (10,000 U/mL penicillin and 10,000 μg/mL streptomycin), in an atmosphere containing 5% CO_2_ at 37 °C [36].

### 2.4. Nanoformulation Preparation Protocols

Nanoemulsions (NEMs) and nanocapsules (QNEMs) were prepared as described previously [34,37] and indicated below.

Nanoemulsions: 30 mg of lecithin was placed in a test tube, to which 2.76 mg of curcumin was added and carefully mixed. Then, 0.5 mL of ethanol was added under constant vortexing until lecithin and curcumin were completely dissolved. Subsequently, 125 μL of Miglyol was added under constant vortexing until a homogeneous solution (organic phase) was obtained. Then, 9 mL of acetone was added to the above mixture. This mixture (organic phase) was added under agitation with a magnetic stirrer to a 50 mL beaker containing 20 mL of Milli-Q water (aqueous phase). This mixture was stirred for an additional 10 min. Then, the organic solvents (acetone and ethanol) were eliminated in a Rotavapor model R-210 (Büchi, Switzerland), reducing the emulsion volume to approximately 4 mL. This emulsion was adjusted to a final volume of 5 mL with Milli-Q water.

Nanocapsules: 30 mg of lecithin was placed in a test tube, to which 2.76 mg of curcumin was added and carefully mixed. Then, 0.5 mL of ethanol was added under vortexing until the lecithin was completely dissolved. Subsequently, 125 μL of Miglyol was added and vortexed until a homogeneous solution (organic phase) was obtained. Next, 9 mL of acetone was added to the above mixture. This organic phase was then added to a 50 mL beaker containing 20 mL of an aqueous solution of chitosan (0.05% *w/v*), under constant mixing on a magnetic stirrer for 10 min. Then, the organic solvents (acetone and ethanol) were removed in the rotavapor until the nanocapsule volume was reduced to 4 mL. Finally, the nanocapsule formulation was adjusted to a final volume of 5 mL with Milli-Q water.

### 2.5. Physicochemical Characterization of Nanoformulations

Nanoformulations were characterized as previously described [34]. In short, the size and zeta potential of colloidal systems were determined via photon correlation spectroscopy and Doppler laser anemometry, respectively. Images were obtained via transmission electron microscopy to analyze the morphology of the nanoformulations. To obtain scanning transmission electron microscopy (STEM) images, a drop (10 μL) of the nanoparticle suspensions was added to a copper grid (mesh 200, covered with Formvar) for 2 min, and the excess aqueous phase of the droplet was carefully removed with filter paper while avoiding touching the grid. Then, grids were washed twice with a drop of Milli-Q water for 1 min, and again, excess water was removed carefully with filter paper. Later, the sample was stained with a drop of 1% phosphotungstic acid for 2 min, and subsequently, liquid was removed with filter paper. Finally, the grid was left to dry for at least 1 h before analysis [38].

### 2.6. Antioxidant Capacity Measurement

The antioxidant capacity (AC) was measured using two different methods, evaluating either the DPPH (1,1-Diphenyl-2-picrylhydrazine) scavenging capacity [39] or the oxygen radical absorbance capacity with fluorescein as the molecular probe (ORAC-FL) [40]. For the DPPH scavenging measurements, 10, 20, 30, 40, and 50 µL of each formulation were added to 990, 980, 970, 960, and 950 µL of a freshly prepared DPPH ethanolic solution with an absorbance of 0.9 units at 517 nm (~50 µM). After 20 min of reaction, the time in which the reaction stabilizes, the solutions were transferred to a 96-well transparent microplate, and the absorbance was evaluated in a Synergy HT multi-detection microplate reader (Bio-Tek Instruments, Winooski, Vermont). Changes in the absorbance at 517 nm were registered for each formulation. The obtained absorbance values were transformed into percentages by means of a direct comparison with the absorbance registered for the blank solution (ethanolic DPPH solution) and then plotted against the sample concentration to calculate the IC_50_ value for each sample. IC_50_ values were then calculated through interpolating inhibition by 50% into the linear regression obtained for each case. Similarly, 20 µL of ethanolic curcumin solutions of 10, 50, 100, 500, and 1000 mgL^−1^ were added to 980 µL of the same DPPH ethanolic solution and measured in the same way as for the formulations to calculate curcumin’s IC_50_.

For the ORAC-FL measurements [40], 10 µL of each formulation sample was diluted in ethanol to a final concentration of 1/15% *v/v* (equivalent to 0.1 µM of curcumin), while for curcumin, an ethanolic solution of 2 mg/L (~5.43 µM) was prepared. Then, 25 µL of each solution was mixed with 150 μL of fluorescein in phosphate buffer of pH 7.4 (40 nM) in a 96-well white polystyrene microplate. Blanks were prepared similarly to the samples but with 25 μL of ethanol instead of the sample. The microplate was placed in a Synergy HT multi-detection microplate reader (Bio-Tek Instruments, Winooski, Vermont) and then incubated for 7 min at 37 °C. The radical reaction was initiated upon adding 25 μL of an 18 mM solution of AAPH (2,2-azobis (2-methylpropionamidine) dihydrochloride) in phosphate buffer, using a multichannel pipette. Then, after gentle shaking, fluorescence in each well was recorded every 1 min for 240 min through measuring the emissions from the top of the microplate (excitation wavelength of 485 nm and an emission wavelength of 520 nm). The kinetics of fluorescence decay was registered in each case, and the resulting area under the curve (AUC) was normalized through subtracting the AUC obtained in the blank experiment. Finally, the normalized AUC values were compared with a Trolox calibration curve (1, 3.5, 5, 10, 30, 50, 60, 80 µM Trolox; r^2^ = 0.999) to determine the µmol of Trolox equivalent per µmol of curcumin (µmol TE/µmol CUR) for each sample.

### 2.7. Minimal Inhibitory Concentration Assay

Blood agar plates were prepared, and 2 mL of the different concentrations of curcumin contained in the nanoformulations or the same volume of their respective vehicles (6.25, 12.5, 25, 50, 75, 100 and 150 μM) were added before solidification. Subsequently, 10 μL of serial dilutions of the cultured bacterium at an optical density of 600 nm were added to the different plates and incubated for 48 h at 37 °C. After that period, the colony-forming units (CFU) were counted.

### 2.8. MTS Viability Assay

A total of 5000 GES-1 cells were seeded per well in a 96-well plate. After 24 h, the cells were treated with different concentrations of curcumin contained in the nanoformulations or their respective vehicles (6.25, 12.5, 25, 50, 75, 100, 150 μM). After 24 h, the medium was retrieved and replaced with fresh medium. MTS reagent prepared according to the manufacturer’s instructions was added to the cells, which were then incubated for 1 h at 37 °C. Finally, the optical density (OD) was measured via spectrophotometry in a plate reader at 490 nm. Viability values were calculated using the following formula: (OD_490_ nm sample × 100)/OD_490_ nm control.

### 2.9. Trypan Blue Viability Assay

A total of 100,000 GES-1 cells were seeded per well in a 24-well plate. After 24 h, the cells were treated with different concentrations of curcumin contained in the nanoformulations or their respective vehicles (6.25, 12.5, 25, 50, 75, 100, 150 μM). After another 24 h, the cells were washed and resuspended in 100 μL of Trypsin-EDTA. Then, 10 μL of this suspension with cells was diluted 1:10 with the trypan blue solution, and viable cells were counted in a Neubauer chamber using an inverted microscope.

### 2.10. Bacterial Internalization Assay

These assays have been described previously [41]. Briefly, 200,000 GES-1 cells were seeded per well in a 6-well plate and incubated at 37 °C/5% CO_2_ for 24 h in RPMI1640 medium supplemented with 10% fetal bovine serum. Then, cells were washed 3 times with PBS and supplemented with medium without antibiotic. Following this, GES-1 cells were infected with *H. pylori* strain 26695 at an MOI = 100 for 6 h. Then, the cells were washed with PBS and exposed to two different conditions: A condition without gentamycin (*w/o* G), in which the total number of bacteria (extra and intracellular) was quantified; and a second condition with gentamycin (with G, 200 μg/mL), in which all the bacteria present in the extracellular environment were eliminated, thereby allowing the quantification of the number of internalized bacteria following cell lysis. Gentamycin was applied at a concentration of 200 μg/mL for 1 h at 37 °C and 5% CO_2_. Then, the cells were washed with PBS. For both conditions, 1 mL of saponin 0.1% was added after the infection. The cells were incubated for 20 min at 37 °C and 5% CO_2_. Finally, supernatant dilutions were prepared and seeded in blood agar plates, which were incubated at 37 °C in microaerophilia for 48 h. After this incubation, the colony-forming units (CFUs) were counted.

The percent of intracellular bacteria was calculated using the following formula: ((number of viable intracellular bacteria) × 100)/number of bacteria associated with GES-1 cells.

### 2.11. Biofilm Formation Assay

These assays were performed as previously described by our group [35]. *H. pylori* was cultured for 3 d in solid medium containing blood agar supplemented with horse serum (5%) and the supplement dent. Then, the optical density of the bacterial cultures was measured, and the bacteria were seeded in a 6-well plate at a concentration of 5 × 10^7^ bacteria/mL in brain–heart infusion (BHI) medium supplemented with 5% horse serum and the Vitox supplement. The plates were maintained for 72 h to favor the formation of biofilms.

After 72 h, the medium was removed, and the plates were washed twice via immersion in distilled water and then left to dry for 20 min in a laminar flow hood. The biofilm formed on the surface of the plate was stained with 1 mL of safranin 0.1% for 15 min. The dye was then removed, and the biofilm was carefully washed to remove the excess dye present on the plate. Subsequently, the dye retained in the biofilm was extracted with 1 mL of 95% ethanol for 5 min and the absorbance was measured at 490 nm.

### 2.12. Biofilm Formation Assays Determined via Counting Colony-Forming Units

*H. pylori* was cultured for 3 d in solid medium of blood agar supplemented with horse serum 5% and the supplement dent. Then, the optical density of the bacteria was measured, and bacteria were distributed in 6-well plates at a concentration of 5 × 10^7^ bacteria/mL in brain–heart infusion (BHI) medium supplemented with 5% horse serum, 1% TSB, and the Vitox supplement. The plates were incubated for 72 h to favor the formation of biofilm.

After 72 h, the medium was removed, and the plates were washed 2 times with PBS and then left to dry for 10 min in a laminar flow hood. The resulting biofilm was then resuspended and carefully recovered in BHI supplemented with horse serum at 5%, 1% TSB, and the Vitox supplement. For each of the conditions, samples were serially diluted and added to blood agar plates supplemented with horse serum 5% and the supplement dent. The colonies were allowed to grow for 48 h, and then CFUs were counted.

### 2.13. Statistical Analysis

All data shown (except for scanning transmission electron microscopy images) are representative of or were averaged from at least three independent experiments. For more details, see the corresponding legends of the tables/figures. Results were analyzed using the ANOVA test (one-way ANOVA). To analyze whether differences were significant, Dunnett’s multiple comparison post-test was used. All values shown were averaged from results obtained in three or more experiments. Differences with *p* < 0.05 were considered statistically significant. All the data obtained were analyzed using the GraphPad Prism program (version 8.0.2). All antioxidant capacity results were analyzed using the ANOVA test (one-way ANOVA). To analyze whether differences were significant, Tukey’s multiple comparison post-test was used. All values shown were averaged from results obtained in three or more experiments. Differences with *p* < 0.05 were considered statistically significant. All the data obtained were analyzed using the GraphPad Prism program (version 8.0.2).

## 3. Results

### 3.1. Nanoformulations Containing Curcumin Are Stable and Show Low Polydispersion Indices

The nanoformulations that were developed and characterized in this study were obtained using the solvent displacement method. This method has been frequently used to obtain nanovehicles with an oily core (oil-in-water formulations), since it allows formulations to be developed simply, quickly, and with low environmental impact [42,43]. The parameters evaluated for the characterization of these formulations were size, zeta (Z) potential, and the polydispersion index (PDI), all important parameters used to characterize the reproducibility and stability of the nanoformulations [34]. In the case of nanoemulsions, the particle size was close to 200 nm with a negative zeta potential of around −60 mV for both the empty vehicle and the nanoemulsions loaded with curcumin (Table 1). Nanocapsules were larger, with a particle size close to 300 nm and a positive zeta potential of approximately +60 mV. Again, similar values were obtained for empty and curcumin-loaded nanocapsules. All nanoformulations (emulsions and capsules) showed a PDI of less than 0.3, indicating populations of particles with low variability in terms of size.

**Figure 1:**
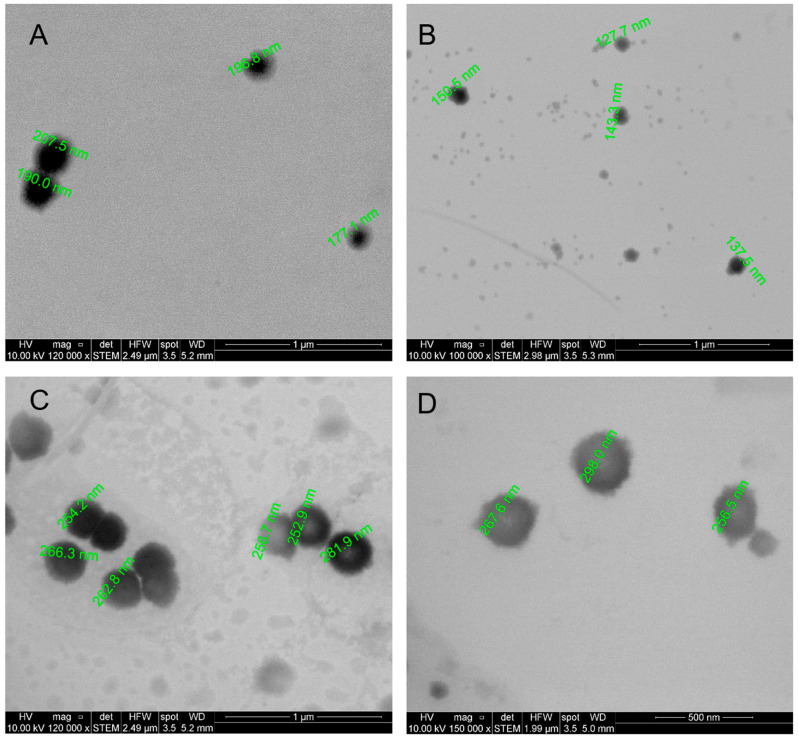
**Scanning transmission electron microscopy (STEM) images.** (**A**) Nanoemulsions with curcumin. (**B**) Nanoemulsions without curcumin. (**C**) Nanocapsules with curcumin. (**D**) Nanocapsules without curcumin. The approximate diameter for some structures was included.

Scanning transmission electron microscopy (STEM) was also performed to characterize the preparations (Figure 1). STEM images show uniform spheroidal structures whose size was smaller than that observed via dynamic light scattering (DLS). These results may reflect differences attributable to the hydration state of nanoformulations in water and dehydration as a consequence of the drying process during sample preparation for STEM.

**Figure 2:**
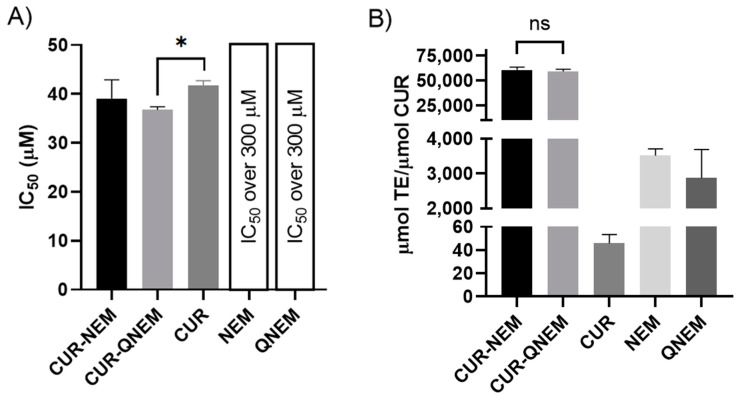
**Antioxidant capacity (AC) of the nanoemulsions and nanocapsules loaded with curcumin (CUR-NEM and CUR-QNEM), curcumin alone (CUR), and the empty nanocarriers (NEM and QNEM)**. (**A**) AC determined via DPPH expressed as IC_50_. Only statistically significant differences are indicated (* *p* < 0.05). (**B**) AC determined via the ORAC-FL method; all possible comparisons except the one indicated as ns were statistically significant (n = 3; *p* < 0.01).

The results shown in Figure 2A are inversely proportional to the AC, meaning that higher IC_50_ values are indicative of lower AC. Taking this into consideration, the CUR-QNEM formulation had the highest AC of the curcumin-loaded formulations, being slightly greater than that of free curcumin. For NEM and QNEM, their IC_50_ values were estimated to be over 300 µM beyond the range that can be determined accurately. To calculate these values, a linear regression was performed between the curcumin concentration in the nanoformulations (µM) and the remanent absorbance. NEM and QNEM did not satisfy this condition, and so their IC_50_ was estimated to be over 300 µM. Figure 2B shows the results obtained using the ORAC-FL assay. In this case, the AC is directly proportional to the obtained values. As shown, no statistically significant difference was found between the AC of CUR-NEM and CUR-QNEM, indicating that both formulations had the same capacity to protect the fluorescent probe against oxygen-centered radicals. Both CUR-NEM and CUR-QNEM nanoformulations had much greater AC values (over 50,000 µmol TE/µmol curcumin) than empty nanoformulations (approx. 3000 µmol TE/µmol curcumin) and free curcumin (46 ± 7 µmol TE/µmol curcumin). With this study, we show that through using nanoformulations to solubilize curcumin, the AC of this compound is dramatically enhanced in a physiologically relevant aqueous phase environment. Considering this key aspect, further studies in this paper were carried out with curcumin included in the nanocarriers. In doing so, biophysical artefacts that may arise upon mixing cells or bacteria with the insoluble curcumin powder were avoided.

### 3.2. Nanoemulsions and Nanocapsules Containing Curcumin Inhibit the Growth of H. pylori 26695

**Figure 3:**
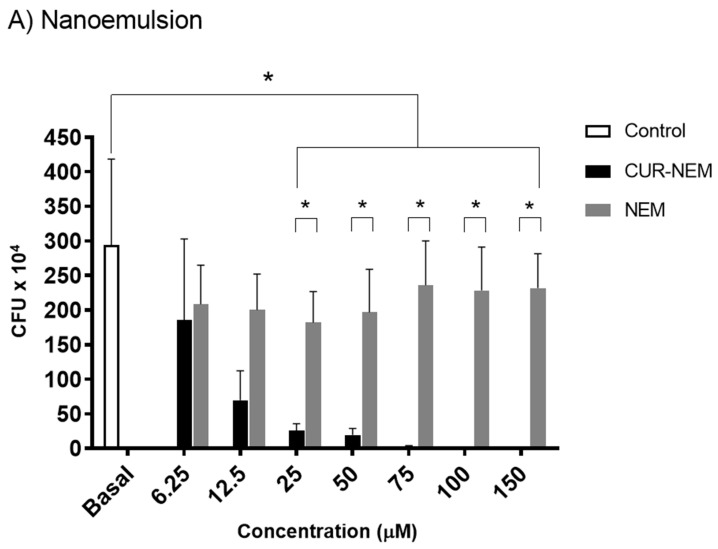

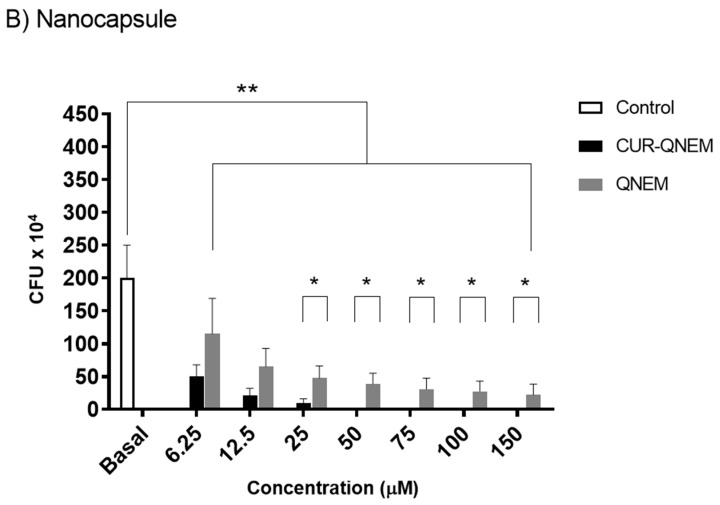
**Inhibition of *H. pylori* 26695 growth by nanoformulations containing or not curcumin**. Results for minimum inhibitory concentration assays (see methods) using nanoemulsions (**A**) and nanocapsules (**B**) either containing or not containing curcumin, which were employed to determine the effect of curcumin on *H. pylori* 26695 growth. The X axis shows the concentration of curcumin (μM) or the equivalent amount of nanovehicle; the Y axis shows the number of bacteria as colony-forming units (CFU × 10^4^). Values shown are equivalent to the mean +/− error from three independent experiments (n = 3). The asterisks indicate statistically significant differences (n = 3; * *p* < 0.01; ** *p* < 0.001).

Since our formulations were characterized and found to be reproducible and stable, and because curcumin has been reported to have antibacterial effects in many studies [18,30,31,32], we evaluated how our formulations affected *H. pylori*. For this, the effect of curcumin contained in nanoemulsions and nanocapsules on *H. pylori* 26695 growth was evaluated in assays designed to determine the MIC. To do so, several concentrations of curcumin in the preparations (from 6.25 μM to 150 μM) were evaluated and compared with the effects of the vehicle alone. For curcumin contained in nanoemulsions, bacterial growth was completely inhibited by a concentration of 75 μM curcumin (Figure 3A), while for the nanocapsule preparations of curcumin, complete inhibition of bacterial growth was observed at 50 μM (Figure 3B). For the nanovehicles without curcumin, the nanoemulsion vehicle had no effect while nanocapsules displayed a significant inhibitory effect on bacterial growth, although complete inhibition was not observed. This effect of the nanocapsules alone was therefore not as pronounced and required higher concentrations than when curcumin was present in the nanovehicles.

#### 3.2.1. Nanoemulsions but Not Nanocapsules Containing Curcumin Reduce GES-1 Cell Viability in a Dose-Dependent Manner

**Figure 4:**
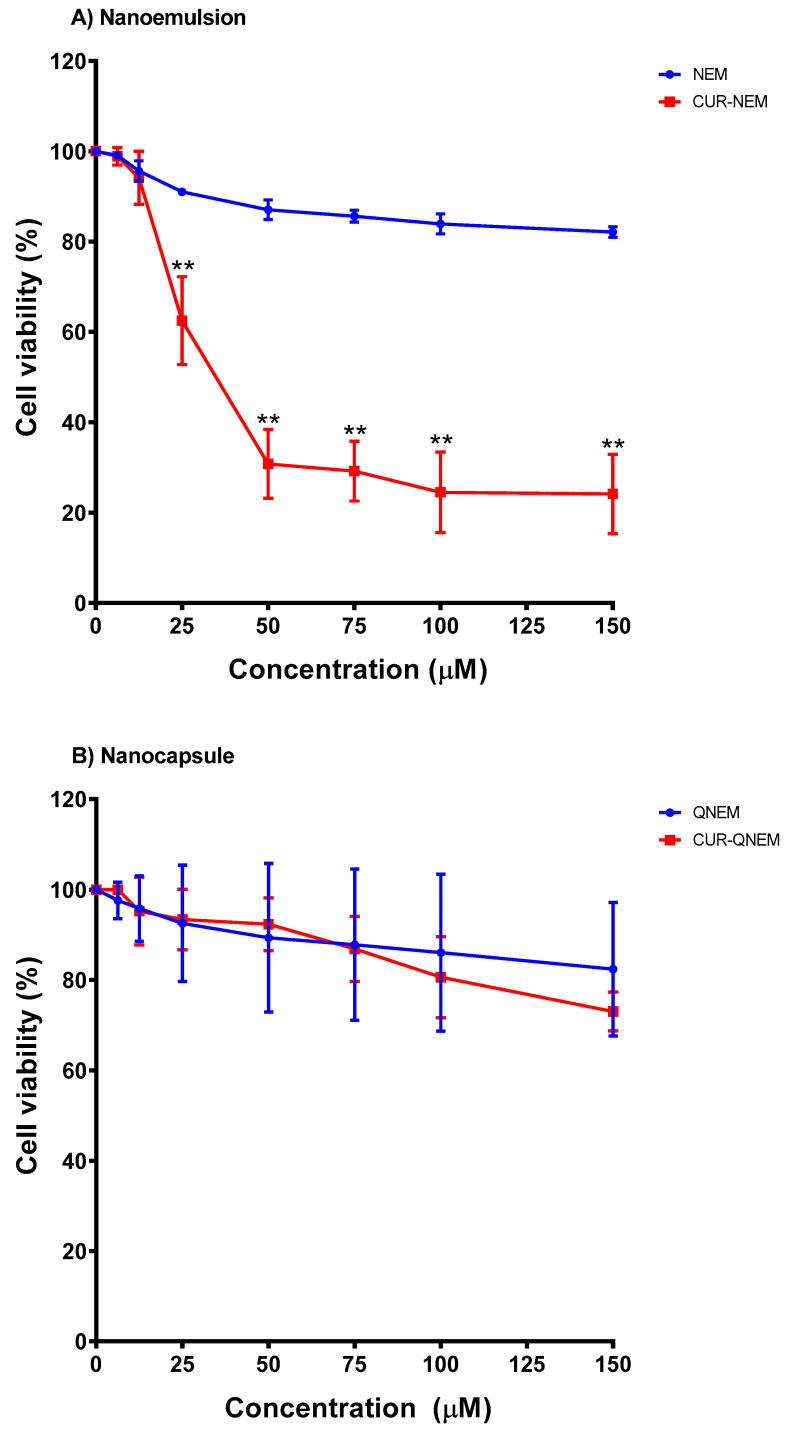
**Cytotoxicity in GES-1 cells of nanoemulsions and nanocapsules containing of not curcumin (MTS assay).** The effects of nanoemulsions (**A**) and nanocapsules (**B**) either containing or not containing curcumin were evaluated in GES-1 cells using the MTS assay. The blue line shows results obtained with the empty vehicle, while for the formulation containing curcumin, results are shown in red. The X-axis shows the concentration of curcumin (µM) or the equivalent amount of nanovehicle alone. Viability is shown on the Y-axis. The results were averaged from three independent experiments, and means +/− error are shown. The asterisks indicate statistically significant differences (** *p* < 0.001) in viability between the control condition with vehicle alone at the various concentrations of curcumin used (n = 3).

Using MTS cell viability assays, the cytotoxicity of curcumin contained in nanoemulsions and nanocapsules was evaluated in GES-1 cells. These cells were treated for 24 h with increasing doses of curcumin contained in the formulations (equivalent amounts of the respective vehicles were also evaluated as controls), and the absorbance at 490 nm was determined. For the nanoemulsions and nanocapsules lacking curcumin, no significant differences were observed compared to the initial viability control (Figure 4A,B). For nanoemulsions loaded with curcumin, a dose-dependent toxicity effect was detected. The highest concentration tested (150 μM) led to a 75% decrease in GES-1 viability (Figure 4A). Indeed, for nanoemulsions loaded with curcumin, statistically significant differences as compared with vehicles (without curcumin) were detected at the concentration of 25 μM and beyond (Figure 4A). On the other hand, for nanocapsules loaded with curcumin, no significant differences were observed in the cell viability, even at the highest concentration (150 μM) (Figure 4B).

**Figure 5:**
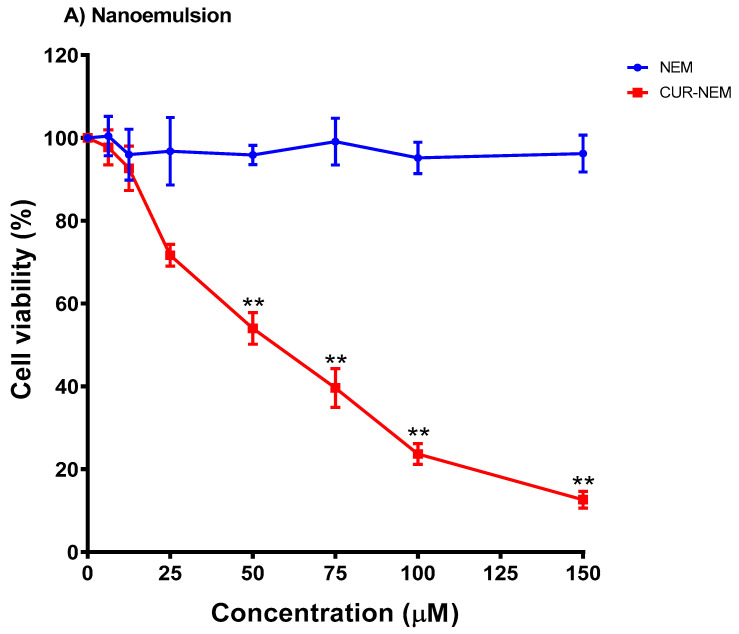

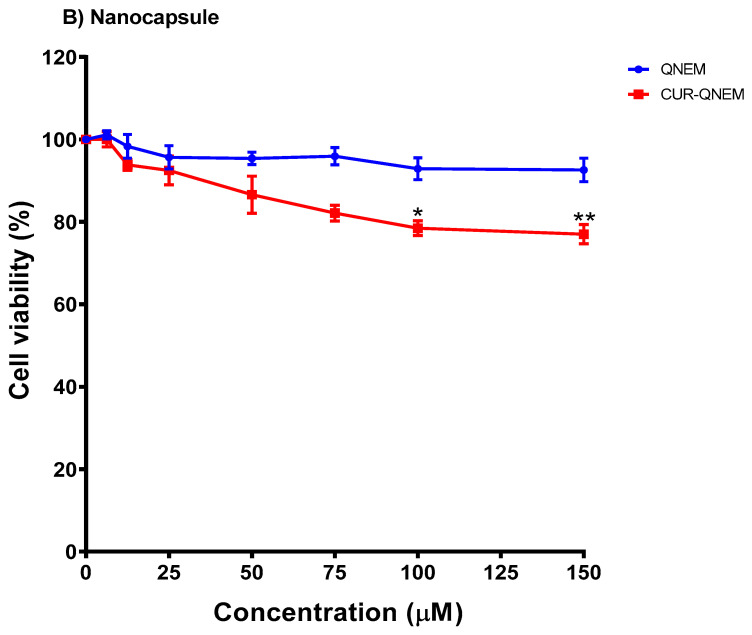
**Cytotoxicity in GES-1 cells of nanoemulsions and nanocapsules containing or not curcumin (trypan blue assay).** The effects of nanoemulsions (**A**) and nanocapsules (**B**) either containing or not containing curcumin were evaluated in GES-1 cells using the trypan blue assay. The blue line shows results obtained with the empty vehicle, while the red line shows those obtained with the formulation containing curcumin. The X-axis shows the concentration of curcumin (µM) or the equivalent amount of nanovehicle alone. Viability is shown on the Y-axis. The results were averaged from three independent experiments, and means +/− error are shown. The asterisks indicate statistically significant differences (* *p* < 0.05; ** *p* < 0.001) in viability between the control condition with vehicle alone and the various concentrations of curcumin used (n = 3).

Additionally, trypan blue assays were performed to complement the MTS cell viability results. The GES-1 cells were treated with increasing concentrations of curcumin contained in the two different formulations. After 24 h, the number of dead cells was determined for all conditions. For empty nanoemulsions, no significant differences with respect to controls were observed for the vehicle (Figure 5A). This result, together with that obtained using the MTS assay (Figure 4A), confirms that nanoemulsions alone are harmless to GES-1 cells. On the other hand, for nanoemulsions containing curcumin (CUR-NEM), a dose-dependent toxicity effect was observed. At a concentration of 50 μM, cell viability decreased by 50%, and at the highest concentration used (150 μM), cell viability decreased by 80% (Figure 5A), following a similar trend to that observed in the MTS assay (Figure 4A).

For empty nanocapsules, no significant differences were observed with respect to the controls for the different concentrations tested (Figure 5B). Thus, together with the results obtained in the MTS assay (Figure 4B), these findings suggest that the nanocapsule vehicle is safe for GES-1 cells at the concentrations assayed. For the nanocapsules containing curcumin, a slight but significant difference to controls was only observed at the highest concentrations tested (100–150 μM). Specifically, at 150 μM, cell viability decreased by a modest 20% (Figure 5B).

Together, the results obtained in the MIC assay and in the cell viability assays using MTS and trypan blue allowed us to define the concentrations to be used for the subsequent experiments (evaluating bacterial adhesion and biofilm formation). For nanoemulsions containing curcumin, the MIC determined was 75 μM; however, at this concentration, we observed a cytotoxic effect in the GES-1 cell model. Therefore, we decided to continue with bacterial adhesion assays using the maximum curcumin/vehicle concentration at which bactericidal toxicity was observed in the absence of detrimental effects for GES-1 cells, namely 12.5 μM for curcumin contained in nanoemulsions. For curcumin in nanocapsules, the MIC determined for *H. pylori* was 50 μM. At this concentration, no cytotoxicity was observed in GES-1 cells. Therefore, this concentration of curcumin/vehicle (50 μM) was used in the subsequent assays.

#### 3.2.2. Nanoformulations Containing Curcumin Diminish the Adherence of *H. pylori* to GES-1 Cells as Well as Internalization of the Bacteria

**Figure 6:**
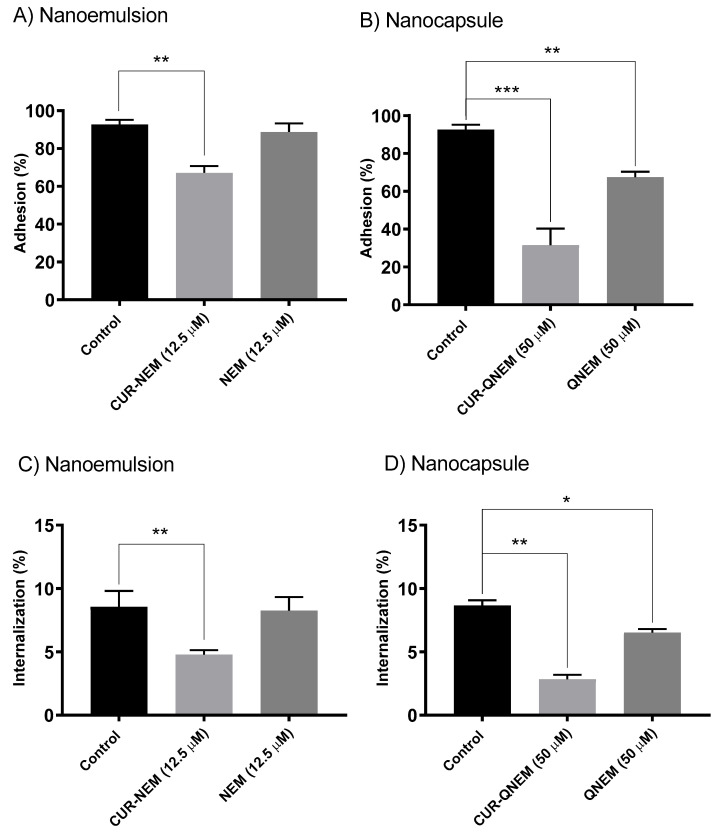
**Curcumin contained in nanoemulsions and nanocapsules decreases the adhesion of *H. pylori* 26695 to GES-1 cells and bacterial internalization**. Adhesion of *H. pylori* 26695 to GES-1 cells was evaluated after preincubation of the cells with either nanoemulsions (**A**) or nanocapsules (**B**) either containing or not containing curcumin for 24 h. Also internalization of *H. pylori* 26695 by GES-1 cells was evaluated after preincubation of the cells with either nanoemulsions (**C**) or nanocapsules (**D**) either containing or not containing curcumin for 24 h. X axis, concentration of curcumin (μM); Y axis, adhesion or internalization of *H. pylori* to GES-1 cells (%). Values shown are the averages from three independent experiments (mean +/− error). The asterisks indicate statistically significant differences (* *p* < 0.05; ** *p* < 0.01; *** *p* < 0.001) between the control condition and the various concentrations used (n = 3).

In the following experiments, we initially sought to determine whether curcumin-loaded nanoformulations were able to prevent the adhesion of *H. pylori* to GES-1 cells. For this, the cells were treated with the curcumin nanoformulations for 24 h prior to infection with *H. pylori*. For curcumin in nanoemulsions at the concentration of 12.5 μM, a significant decrease of 30% was observed for bacterial adherence to GES-1 cells (Figure 6). For the equivalent concentration of the vehicle alone, no significant decrease in bacterial adhesion with respect to the controls was detected (Figure 6). Importantly, at this concentration of curcumin in nanoemulsions, internalization was reduced by 50%, while the vehicle alone had no effect. For the curcumin in nanocapsules at a concentration of 50 μM, a 65% decrease in bacterial adherence to GES-1 cells as compared to controls was detected. In this case, the vehicle alone also decreased bacterial adherence to the GES-1 cells by 30%, when compared to the control situation (Figure 5). Likewise, the curcumin-loaded nanocapsule formulation at this concentration reduced internalization by 70%, and in this case too, the vehicle alone reduced internalization by 25% (Figure 6).

#### 3.2.3. The Nanoformulations Reduce Biofilm Formation by *H. pylori*

**Figure 7:**
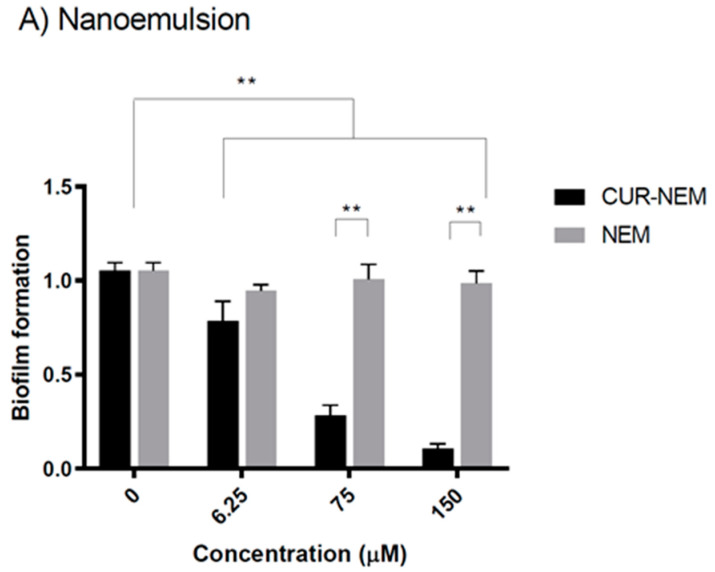

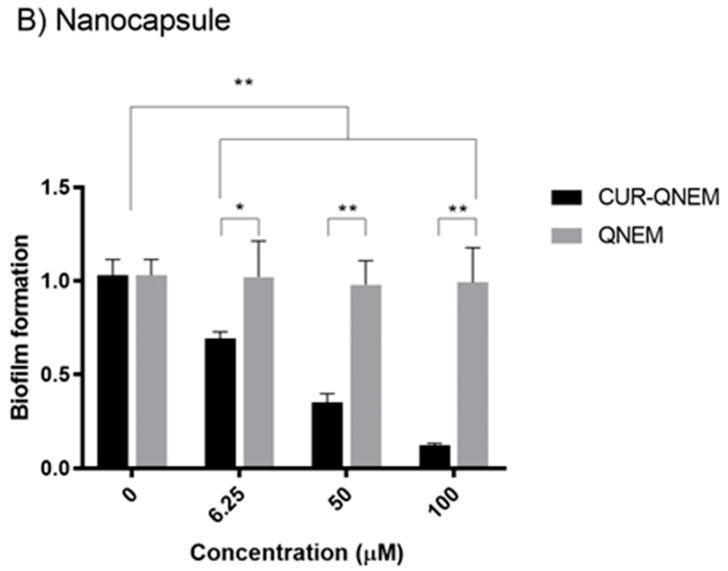
**Curcumin in nanoemulsions and nanocapsules decreases biofilm formation by *H. pylori*.** *H. pylori* biofilm formation assays were performed in the presence of either curcumin nanoemulsions (**A**) or nanocapsules (**B**). X axis, concentration (μM); Y axis, biofilm formation (fold change) by *H. pylori* 26695. Values shown are the averages from three independent experiments (mean +/− error). The asterisks indicate statistically significant differences (* *p* < 0.05; ** *p* < 0.001) with respect to the control condition (n = 3).

Biofilms are organized structures that permit interaction between bacteria. Hence, it is an important strategy employed to enhance intercellular communication between bacteria and increase survival [44]. To extend our analysis of the effects of nanoformulations on *H. pylori* 26695, the bacteria were incubated with three different concentrations of nanoformulations, a concentration at which there were no significant effects on bacterial growth (6.25 μM), the respective minimum inhibitory concentrations for each formulation (75 μM and 50 μM), and twice the MIC in each case (150 μM and 100 μM). For the curcumin nanoemulsions, no statistically significant effect at the lowest concentration of 6.25 μM was observed (Figure 7A); however, when the MIC was tested, we observed a 50% decrease in biofilm formation as compared to the control. Moreover, when using twice the MIC, a decrease in biofilm formation of about 80% was observed (Figure 7A). In both cases, the nanoemulsion vehicle alone had no significant effect.

For the curcumin-loaded nanocapsule formulation, the lowest curcumin concentration tested (6.25 μM) had no effect on biofilm formation (Figure 7B); however, when using the MIC, we observed a 50% decrease, and with twice the MIC, a decrease in biofilm formation of 90%, as compared to controls (Figure 7B). In this case, the nanocapsule vehicle alone had no significant effect.

## 4. Discussion

In the present study, a nanoemulsion and a nanocapsule formulation, capable of entrapping curcumin in the oil core and being dispersible in water/biological media, were prepared and characterized, and subsequently, their effects against *H. pylori* were evaluated. We observed that both nanoemulsions and nanocapsules have diameters of around 200 nanometers, with low polydispersion and zeta potential values consistent with them being stable over time and reproducible from batch to batch. Then, MIC assays were performed to determine the antibacterial potential of curcumin in such preparations against *H. pylori*. The MIC is defined as the lowest concentration at which an antibacterial agent is capable of completely eliminating bacterial growth [45]. The effects of curcumin against various bacterial agents have been evaluated previously. However, generally, in such experiments the vehicle for curcumin was water, ethanol, or DMSO. For the curcumin extracts in water, an MIC in the range of 4 to 16 g/L was obtained against the following bacteria: *S. epidermis ATCC 12228, Staph. aureus ATCC 25923, Klebsiella pneumoniae ATCC 10031,* and *E. coli ATCC 25922* [46]. As mentioned above, curcumin is poorly soluble in water; therefore, large doses of curcumin are required to inhibit the growth of the aforementioned bacteria. Our formulations are freely dispersible in the aqueous phase thanks to possessing a polar surface, while curcumin is dissolved in the oily core, where it is stabilized and protected. Thus, notably lower concentrations of curcumin in our nanoformulations are effective at decreasing *H. pylori* growth.

On the other hand, curcumin can be solubilized in organic solvents like ethanol and, as such, decreases the growth of *Bacillus subtilis* and *Staphylococcus aureus* at concentrations of 16 μg/mL and 128 μg/mL, respectively [47]. However, in this case, the preparations were applied topically to HIV patients to prevent itching syndrome and wound infection. Being topical applications, the use of organic solvents is not necessarily a problem, mainly because the solvent evaporates after the application. Alternatively, however, the use of alcohol and other organic solvents is highly restricted for orally administered medicines, nutraceutical formulations, and food products.

The use of several formulations to stabilize curcumin has been widely studied as a means to protect the molecule against oxidation while enhancing its bioavailability due to low water solubility [48]. Some of these studies have investigated the antioxidant capacity of curcumin in different nanoformulations. In particular, Choi et al. studied curcumin and quercetin loaded into freeze-dried mushroom microparticles (FDMMs). Both antioxidants were able to diminish lipid peroxidation in food products and thereby aid in their preservation [49]. In this study, the authors also measured FDMMs via DPPH and reported that the AC (IC_50_ values) for both polyphenols was lower than the value for polyphenol-loaded FDMMs, which is similar to the results shown in Figure 2A. Similarly, Feng et al. also reported an increase in the AC determined using the ABTS• method when curcumin or proanthocyanidins were included in nanoparticles containing Fe^2+^ [50]. This is especially important when measuring the AC using the ORAC-FL method, as the reaction medium is mainly water. Due to the very low water solubility of curcumin, it is likely that even at the tested concentration (approximately 0.67 µM), not all curcumin was dissolved and available to develop its AC, leading to the low values detected using this method (Figure 2B). This point reveals again the importance of carriers to improve curcumin’s water solubility to allow it to develop its optimal chemical potential in biological systems.

To the best of our knowledge, most of the scientific studies concerning curcumin stabilization also include another polyphenolic compound, and so, the activity of the nanoformulation is thought to derive from the synergy of two polyphenolic compounds [48]. With this study, we show that through using nanoformulations to solubilize curcumin, the AC of this compound is dramatically enhanced in a physiologically relevant aqueous phase environment.

In the present study, MIC values of 75 µM or 27.6 µg/mL for the nanoemulsions and of 50 µM or 18.5 µg/mL for the nanocapsules containing curcumin were obtained. These results are consistent (especially the value for nanocapsules) with previous studies reporting MIC values against *H. pylori* of 18 µg/mL [32] and 19 µg/mL for curcumin dissolved in DMSO [33]. Thus, the novelty of this study resides in showing that our nanosystems containing curcumin are as effective at eliminating *H. pylori* as when the compound is solubilized in organic solvents. As such, they represent safe and stable systems containing curcumin as an active agent that can be used to eliminate *H. pylori*. For the nanocapsule formulations, the cytotoxicity against *H. pylori* was even more significant. This is most likely due to the presence of chitosan, since this polymer, apart from its mucoadhesive properties, reportedly also has antimicrobial activity [33]. Chitosan can bind to the bacterial wall, which generates a change in membrane permeability, that allows chitosan to enter the bacteria and bind to bacterial DNA, which then impedes replication and induces bacterial death [51]. Another possible mechanism that may explain the effects of chitosan is that it acts as a chelator which can bind to trace metals present in the medium and thereby reduce the production of bacterial toxins and other elements that inhibit microbial growth [52,53]. However, inhibition of *H. pylori* growth was more significant upon the inclusion of curcumin. Thus, curcumin as a nanoemulsion reduces *H. pylori* growth, but together with chitosan in nanocapsules, the effect is more significant.

Beyond the cytotoxic effects on *H. pylori*, it was also important to determine whether these formulations may affect gastric cell viability at the concentrations employed. To this end, cell viability was assessed in MTS (Figure 4) and trypan blue (Figure 5) assays. Rather unexpectedly, based on our previous studies using mammalian cells, the nanoemulsions showed some toxicity towards human gastric GES-1 cells, whereby cell viability decreased by 60% at concentrations equivalent to the MIC values for *H. pylori*. The result generated a difficulty for the subsequent experiments evaluating bacterial adherence because it was not possible to compare both nanoformulations using the previously defined MIC for *H. pylori*. Due to this situation, the effects of curcumin nanoemulsions were evaluated at the concentration of 12.5 µM, which is the maximum concentration at which no significant effect on GES-1 viability was observed. For the nanocapsules, the MIC was employed since there was no significant impact on GES-1 viability. Interestingly, even using only 12.5 µM of the curcumin nanoemulsion, a significant 30% reduction in bacterial adhesion to GES-1 cells after 24 h was observed. For nanocapsule preparations with curcumin, an approximately 60% reduction in bacterial adhesion to GES-1 cells was observed. This significant difference between the nanoemulsion and the nanocapsule is likely due to use of the MIC for curcumin contained in the nanocapsules but not for the nanoemulsions. However, it is important to highlight that although the MIC was not used in the case of the nanoemulsions, the concentration used significantly reduced bacterial adhesion to GES-1 cells.

Precisely how curcumin elicits such effects is not clear. However, curcumin administered as a nanoemulsion is taken up and accumulates in mammalian cells as early as 2 h after incubation [34]. *H. pylori* has been shown to increase NF-κB p65 and pro-inflammatory cytokine levels in cells [54]. Due to the sustained presence of the bacteria, inflammation becomes chronic and leads to the destruction of gastric tissue. Alternatively, the same study showed curcumin exposure decreases NF-κB p65 expression and reduces the levels of pro-inflammatory cytokines, thereby blocking the pro-inflammatory effects of *H. pylori*. Here we showed that in the presence of nanocapsules with curcumin, cell adhesion and the subsequent internalization were more significantly reduced. Since chitosan is mucoadhesive, the exacerbated effects on *H. pylori* adherence may result from the combination of a physical impediment of the interaction and the blocking of intracellular signaling pathways. Moreover, one may predict based on the aforementioned discussion that the secretion of pro-inflammatory cytokines from infected cells should be reduced by curcumin nanocapsules. While other intriguing possibilities also exist, additional studies will be required to elucidate the mechanisms responsible for the effects observed here and their consequences in detail.

An important process for *H. pylori* colonization of the gastric epithelium involves the contact with and adhesion to the epithelium to obtain the necessary nutrients to survive [9]. Bacterial adhesion to the epithelial cells is also the first step in the colonization and biofilm formation in the stomach. Additionally, biofilm formation increases bacterial resistance to antibiotics and aids in chronically establishing their niche [55]. It has been observed that curcumin, in addition to inhibiting the formation of the Z ring, can inhibit the quorum-sensing activity of bacteria, not through killing the bacteria themselves or through destroying the biofilm that the bacteria form when colonizing a tissue, but through exerting an inhibitory effect on the biofilm formation process [56]. Furthermore, curcumin prevents adhesion and biofilm formation via modulating the quorum-sensing system of bacteria [56]. Our results show that curcumin nanocapsules and nanoemulsions significantly decrease the biofilm formation ability of *H. pylori*, which, as pointed out, is highly relevant to bacterial pathogenesis. Finally, curcumin inhibits the virulence factors that depend on this system [57]. Indeed, it has been observed that curcumin inhibits biofilm formation of the *H. pylori* strains ATCC43504, ATCC43526, and ATCC51932 and, in turn, is able to reduce *H. pylori* adhesion to HEp-2 cells [33]. Rather intriguingly, we observed that both curcumin nanoemulsions and nanocapsules prevent biofilm formation very effectively.

In summary, the results of this study confirm the potential utility of curcumin nanoformulations as biomedical tools to eliminate *H. pylori*. The novelty of these findings resides in the fact that the curcumin was solubilized and made compatible with the aqueous phase using nanotechnology that also protects this highly sensitive molecule against the detrimental effects of pH (important in the stomach), light/UV exposure, and oxidation [34]. Moreover, such preparations also reduce the ability of *H. pylori* to seek refuge in the intracellular environment of host cells where the bacteria are protected from antibiotics. Finally, we also provide evidence indicating that the nanocapsule presentation of curcumin was more effective through several mechanisms: inhibiting *H. pylori* growth, decreasing adhesion and the subsequent internalization into gastric cells, and reducing the biofilm formation ability. Moreover, for nanocapsules, no toxicity was observed in gastric cells. These findings highlight the therapeutic potential of nanocapsule-based curcumin preparations in particular to eliminate *H. pylori* or for use as an adjuvant in therapies against *H. pylori* based on treatments with antibiotics. In addition, considering that one of the main risk factors associated with the development of gastric cancer is the presence of *H. pylori* in the stomach, our formulations will likely be extremely useful for preventing this disease.

Thus, the studies reported on here uncover a rather unique potential particularly for those nanoformulations of curcumin, obtained using nanocapsules, to be employed to reduce or eliminate *H. pylori* in human patients, possibly even without the necessity of employing antibiotics, given that the development of resistance is increasingly becoming a problem in the clinic. Despite this possibility, we do not discard the use of these nanocarriers to complement current therapies or new potential treatments (based on compounds of natural or synthetic origin) to synergistically treat *H. pylori.* However, a clear limitation to these studies is that this potential was documented here in in vitro studies, infecting gastric cells with *H. pylori* in culture or treating the bacteria directly with the formulations. This represents a limitation of our current study, and in the future, we will initially seek to evaluate how diet supplementation with these formulations in mice may help eliminate bacteria in the stomach of infected animals. Thereafter, we envisage extending such studies to humans, looking at how oral administration of these nanoformulations to patients with *H. pylori* infection may help reduce or even eliminate bacteria present in the stomach. Clearly, given our in vitro results, we anticipate a bright future in this respect for nanocapsules containing curcumin.

## 5. Conclusions

Curcumin is an attractive candidate to treat *H. pylori* infection, but its low solubility in water and high instability represent major challenges that need to be addressed when proposing the efficient application of formulations containing this molecule. In this study, we developed nanoemulsions and chitosan nanocapsules loaded with curcumin to maintain/promote the activity of curcumin against this bacterium. In the aqueous phase, the nanoemulsions and capsules demonstrated antioxidant capacity far superior to non-processed curcumin. The nanoemulsions had an average diameter of 200 nm, a PdI ≤ 0.16, and a negative zeta potential (−54 mV), while nanocapsules were on average 305 nm in diameter, with a PdI ≤ 0.29 and a positive zeta potential (+68 mV). The systems were tested in terms of safety (MTS and trypan blue assays) using gastric cells (GES-1), whereby the nanocapsules were found to be safer than nanoemulsions. In terms of efficiency in inhibiting *H. pylori* growth, the MICs for nanoemulsions and nanocapsules were 75 μM and 50 μM, respectively. In the latter case, the presence of chitosan contributed significantly to the inhibitory effect on bacterial growth. Both formulations were effective at preventing the infection/colonization of GES-1 cells by the bacterium. Finally, the formulations also reduced biofilm formation by *H. pylori*, which is a key parameter associated with the prolonged survival and resistance of the bacterium to antibiotics. Considering the simplicity of preparation of these formulations and their demonstrated activity against *H. pylori* at different biological stages, we propose these formulations have great potential to treat or complement current therapies used for *H. pylori* eradication.

## Figures and Tables

**Table 1:** **Characterization of the formulations:** CUR-NEM*: nanoemulsions containing curcumin; NEM*: nanoemulsions without curcumin; CUR-QNEM*: nanocapsules containing curcumin; QNEM*: nanocapsules without curcumin. Preparations were characterized via DLS and laser Doppler anemonotropy (LDA) (n = 3).

Nanoformulation	Diameter (nm) D.E	Polydispersion Index + D.E	Zeta Potential (mV) + D.E
CUR-NEM*	202 ± 3.0	0.16 ± 0.06	−54.2 ± 1.2
NEM*	207 ± 0.3	0.14 ± 0.03	−56.3 ± 0.4
CUR-QNEM*	305 ± 7.0	0.29 ± 0.03	67.8 ± 0.96
QNEM*	296 ± 4.1	0.29 ± 0.02	66.8 ± 0.84

## Data Availability

Data is contained within the article.
